# Hepatic perivascular epithelioid cell tumor: Case report and brief literature review

**DOI:** 10.1097/MD.0000000000005572

**Published:** 2016-12-23

**Authors:** Da Tang, Jianmin Wang, Yuepeng Tian, Qiuguo Li, Haixiong Yan, Biao Wang, Li Xiong, Qinglong Li

**Affiliations:** a Department of General Surgery, Second Xiangya Hospital, Central South University; b Department of General Surgery, the First Hospital of Hunan University of Chinese Medicine, Changsha, Hunan, P.R. China.

**Keywords:** liver, neoplasm, PECmoa, perivascular epithelioid cell tumor, surgery

## Abstract

**Rational:**

Perivascular epithelioid cell tumor (PEComa) is a rare mesenchymal neoplasm which expresses both myogenic and melanocytic markers. PEComas are found in a variety locations in the body, but up to now only approximately 30 cases about hepatic perivascular epithelioid cell tumor are reported in English language worldwide.

**Patient concerns:**

A 32-year-old woman was admitted in our hospital with intermittent right upper quadrant pain for 1 month and recent (1 day) progressive deterioration.

**Diagnoses:**

Based on the results of the laboratory examinations and the findings of the computed tomography, the diagnosis of hepatic hamartoma or the hepatocecullar carcinoma with hemorrhage was made.

**Interventions:**

The patient underwent a segmentectomy of the liver, and the finally diagnosis of hepatic PEComa was made with immunohistochemical confirmation with HMB-45 and SMA.

**Outcomes:**

There is no clinical or radiographic evidence of recurrence 9 months after surgery.

**Lessons:**

This kind of tumor is extremely rare and the natural history of PEComa is uncertain, as the treatment protocol for hepatic PEComa has not reached a consensus. But the main treatment of the disease may be surgical resection. Only after long term follow-up can we know whether the tumor is benign or malignant. It appears that longer clinical follow-up is necessary in all patients with hepatic PEComas.

## 1 Introduction

In 1992, perivascular epithelioid cells were first proposed by Bonetti et al,^[1]^ whereas the term perivascular epithelioid cell tumor (PEComa) was introduced by Zamboni et al in 1996.^[2,3]^ In 2002, the World Health Organization defined PEComa as unusual mesenchymal tumors which compose of histologically and immunohistochemically distinctive perivascular epithelioid cells.^[4]^ The PEComa tumor family includes classic epithelioid angiomyolipoma (AML), lymphangioleiomyomatosis, pulmonary and extrapulmonary clear-cell “sugar” tumors, and PEComa.^[5–8]^ PEComas can arise from many locations of the body, but only a small number of studies reported both benign and malignant PEComa of the liver. The cases of the hepatic PEComas are uncommonly with the information of the hepatic PEComas are not well-described. They are difficult to diagnose before operation or without a biopsy. There are no guidelines concerning diagnostics and follow-up. We analyzed clinical manifestation, imaging characteristics, histopathological findings, and treatment in patients with hepatic PEComas.

## 2 Case report

A 32-year-old woman was admitted in our hospital with intermittent right upper quadrant pain for 1 month and recent (1 day) progressive deterioration. The past medical history of the patient was unremarkable. Physical examination revealed upper abdominal tenderness and mild hepatomegaly without palpable mass. The laboratory examinations showed that the routine blood test, routine stool test, and routine urine test were normal. The level of total protein, albumin, globulin, alanine aminotransferase, aspartate aminotransferase, blood urea nitrogen, serum creatinine, carbohydrate antigen 19–9 (Ca199), carcinoembryonic antigen (CEA), and the alpha-fetoprotein (AFP) were within the normal limits. Hepatitis B and hepatitis C virus tests were negative.

**Figure 1:**
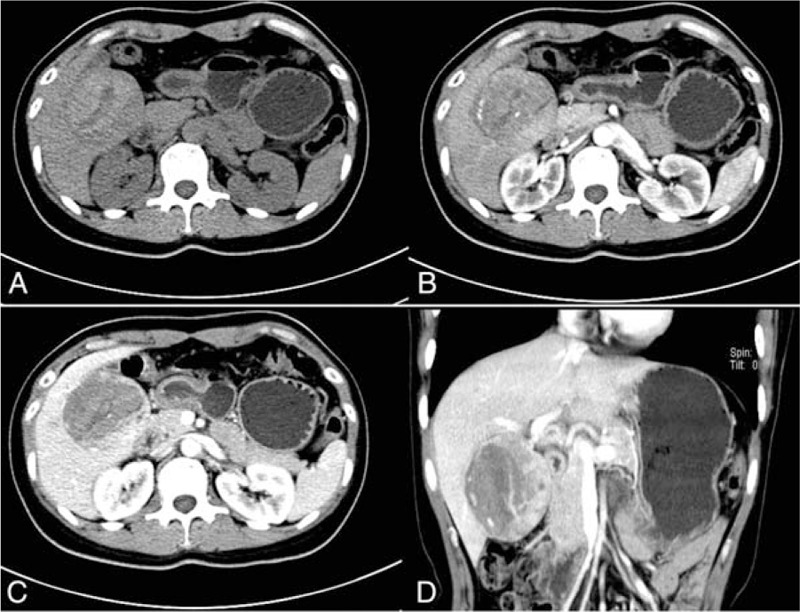
Computed tomography (CT) images of the tumor. A, Plain CT scan showed a round mixed density lesion with well-demarcated margin in the segment V. B, Contrast-enhanced CT showed a marked inhomogeneous enhanced lesion in the arterial phase. C, Contrast CT scan of the same lesion showed the lesion still enhanced obviously in the portal phase. D, Three-dimensional reconstruction of the liver mass on CT.

The plain computed tomography (CT) scan showed a round mixed density lesion with well-demarcated margin, sized 6.5 × 6 × 6 cm, in the segment V of the right lobe of the liver (Fig. 1A). Contrast-enhanced CT showed it was inhomogeneous enhancement on the early arterial phase (Fig. 1B), and the lesion remained obvious heterogeneous enhanced in the portal phase (Fig. 1C). The diagnosis of the CT was hepatic hamartoma or the hepatocecullar carcinoma with hemorrhage. The patient suffered abdominal pain and the malignant tumor could not be ruled out, so the right V segmentectomy of the liver was performed in our hospital.

During the operation, we observed extensive adhesions between the tumor and the surrounding tissues, as both of them appeared hyperemia and edema. After carefully separating the adhesions, we removed the tumor step by step with Pringle maneuver. The resected specimen demonstrated a 7 × 6 × 5 cm solitary tumor with a clear margin. The external surface of the mass was white-tan to gray-red. The mass was soft, smooth, and fragile with multiple congested blood vessels. Hemorrhage was observed in part of the neoplasm, and the amount of bleeding in surgery was about 300 mL.

**Figure 2:**
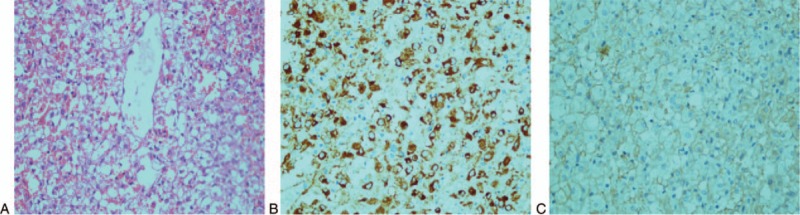
Histological and immunohistochemical pictures of the lesion. A, Histopathology showed that there were a majority of epithelioid and spindle-shaped cells with oval nuclei and clear to granular eosinophilic cytoplasm (magnification 400×). B, Immunohistochemistry showed that the lesions were positive for HMB-45 (magnification 400×). C, Tumor cells showing strong positive staining for SMA (magnification 400×).

On microscopic examination, there was a majority of epithelioid and spindle-shaped cells with oval nuclei and clear to granular eosinophilic cytoplasm. Necrosis, cellular atypia, and nuclear atypia had not been seen (Fig. 2A). The tumor on immunohistochemistry showed that the lesions were positive for human melanin black-45 (HMB-45) (Fig. 2B), smooth muscle actin (SMA) (Fig. 2C), CD99, vimentin, and CD34, but the CD117, S100, CK, CK18, and Ki-67 immunohistochemistry outcomes were negative.

The correct diagnosis of hepatic PEComa was made on the basis of morphologic characteristics and immunohistochemical results. The patient recovered uneventfully and was discharged 1 week after surgery. There is no clinical or radiographic evidence of recurrence 9 months after surgery.

## 3 Discussion

Perivascular epithelioid cell tumors can occur at any age, and the median age is 43 years, but some studies have shown that women are in a strong predominance.^[2,9–11]^ Hormones may play a key role in the pathophysiology. However, The histogenesis and pathogenesis of perivascular epithelioid cells (PECs) are still uncertain because physiological counterpart of PECs have not been defined.^[2]^ There are some hypotheses relating to the origin of PEC. The first one is that the PEC may develop from undifferentiated neural crest cells which have the ability of co-expressing the phenotype of smooth muscles and melanocytes. Another suggestion is that that the PECs may derive from local pericyts. And finally, PECs may have smooth muscle origin at the molecular level which brings about expression of melanocyte markers and melanogenesis.^[12,13]^

In a word, the origin and function of PEComas system still need to be further studied. More and more reports have demonstrated different anatomical sites of these lesions, including broad ligament, retroperitoneum, mediastinum, nasopharyngeal cavity, buccal mucosa, abdominal wall, skin, spinal cord, duodenum, ileum, jejunum, colon, rectum, ligamentum teres and falciform ligament, common bile duct, pancreas, bladder, prostate, penis, breast, uterus, cervix, vulva, ovary, heart, lung, kidney, base of skull, urinary bladder, and pelvic wall.^[14,15]^

Clinical manifestations of a hepatic PEComa is minimal, and it is often found accidentally in physical examinations. Some patients have gastrointestinal symptoms, such as abdominal pain, abdominal discomfort, abdominal distress, nausea, upper abdominal mass, and vomiting, and the reason is that the lesions grow in size and cause localized pressure effect or stretching of liver capsule.^[16,17]^ Even patients with small tumors may also suffer clinical symptoms. For the hepatic PEComa, it is difficult to differentiate a benign tumor from malignant variant only in clinical manifestations. So the imaging findings and histological evaluation might provide valuable diagnostic information.

Imaging diagnosis can also be challenging because the quantity of adipose tissue, irregular vessels, and smooth muscle cells is various. Reviewing the literatures, we can see that the features of ultrasonography are diverse in different people. The hepatic PEComas can be revealed as hyperechoic mass,^[6,18]^ isoechoic tumor,^[19,20]^ or hypoechoic neoplasm.^[9,16,21,22]^ Color Doppler flow image showed all tumors were hypervascular. When injecting a sulfur hexafluoride microbubble as a contrast medium of the enhanced ultrasonography, the lesions appeared clearly hypervascular during the arterial phase and isoechoic in the portal phase, and hyperechogenicity in the equilibrium phase.^[18]^

The precontrast CT of our patient showed partial high density in the tumor. For most patients, the lesions exhibited mass with heterogeneous low density in plain CT^[5,9,16,21]^; at the same time, the tumor can be well-demarcated or ill-defined. But for 1 patient, nonenhancement CT scans revealed a huge cystic tumor in the right lobe of the liver.^[23]^ Contrast-enhanced CT shows almost all hepatic PEComas were hyperenhanced in the arterial phase. In the portal venous phase, the lesions can present as a hyperenhanced, isoenhanced, or hypoenhanced tumor.^[22]^ In the delayed phase, the density of the neoplasms returned to an hypoenhanced or isoenhanced state.^[22]^ According to the literatures,^[3,5,6,18,20,21,24,25]^ we can get that the magnetic resonance imaging (MRI) to most of tumor appeared hypointensity on T1-weighted images (T1WI) and hyperintensity on T2-weigthed images (T2WI). After injecting gadolinium as a contrast medium, the lesions presented a strong enhancement in the arterial phase, as this enhancement was obviously attenuated during portal venous phase and hypointense appearance was observed in the late parenchymal phase.^[6,18]^ Angiography showed that the tumor was abundant of thickened and distorted vessels.^[16,26]^

The final diagnosis of PEComa depends on pathology and immuohistochemistry.^[4,27]^ PEComas are characterized by their perivascular location, and the cells are with radial arrangement around the vascular lumen. Typically, cells around the vessels are epithelioid and spindle-shaped, resembling smooth muscles cell, and have abundant clear to eosinophilic granular cytoplasm.^[20,28]^ Hemorrhage and necrosis can be seen in the malignant hepatic PEComa.^[29]^ Positive immunostaining for melanocytic markers (HMB-45 and/or melan-A) and smooth muscle (actin and/or desmin) are the most histological findings. In the present case, the tumor cells were positive for HMB-45 and SMA, which is the key point of the final diagnosis.

Although the vast majority of reported hepatic PEComas showed a benign course, malignant tumors have also been reported.^[2,29]^ In 2005, Folpe et al^[30]^ reviewed 26 cases of gynecological origins and soft tissue, and proposed a classification of PEComas into benign, semimalignant, and malignant based on the 7 worrisome features of the histology: a tumor size >5 cm; infiltration into the surrounding normal tissue; high nuclear grade; high cellularity; motic activity of more than 1/50 per high power field; coagulative necrosis of the tumor; vascular invasion. The PEComa with 2 or more of the features that are listed should be considered to be malignant. Tumors with nuclear pleomorphisms only or tumors of size more than 5cm only are considered as neoplasms of uncertain malignant potential. Our case exhibited 1 adverse condition that favored semimalignant potential, tumor size >5 cm, so requiring a long-term follow-up.

The treatment protocol for hepatic PEComa has not reached a consensus. Because it is regarded as tumors with uncertain biological potential, most of the neoplasm were removed by surgery. Keeping enough margin during the surgical resection is considered as the gold standard for the treatment of hepatic PEComa.^[31]^ At the same time, except for surgery, radiotherapy, chemotherapy and targeted therapy have also been suggested.^[32]^ In some references, it showed that chemotherapy and radiotherapy do not improve the survival time of the patients.^[5,13]^ However, the promising results of both radiation therapy and chemotherapy are published in a recent review of 234 cases of PEComa.^[10]^ It proved that using chemotherapy or chemoradiation with response rates from 0% to 80% and receiving radiation alone was no response, as using chemotherapy, radiation, imatinib, and mammalian target of rapamycin (mTOR) inhibitors to the metastatic disease had shown variable responses.^[3,10]^ PEComas express p70S6K, which is responsible for the regulation of the mTOR signaling pathway.^[3]^ Several patients of metastatic PEComas were treated with mTOR inhibitors, such as sirolimus or everolimus, which were sensitive to the tumors.^[3]^ A 31-year-old woman was diagnosed with a large and aggressive variant of a hepatic PEComa through biopsy. The neoplasm was so big and close to the hepatic vein, but it showed obvious shrinkage after upfront treatment with mTOR inhibitor sirolimus for 8 months. Thereafter, the mass had been radical removed without complications.^[24]^ It indicates that chemotherapy, combined with surgery, can achieve a good outcome.

## 4 Conclusion

Hepatic PEComas are rare but increasingly recognized tumors. Still, there is a curiosity, and the diagnostic approach, treatment modalities, and the follow-up are faced with challenge. The final diagnosis of PEComa is dependent on histological findings and immuohistochemical characteristics such as HMB-45 and melan-A. Although every neoplasm in the liver cannot be identified by radiologic imaging, various examinations, including ultrasound, CT, and MRI, can still provide significant resources to the doctors. Whether the tumor is benign or malignant could not be predicted, and the main treatment of the disease may be surgical resection. It is necessary to save adequate margin during the operation. Radiotherapy, chemotherapy, and immunotherapy are also the various ways of treatment. If tumor size is less than 5 cm, we can take a wait-and-see attitude, and surgical intervention might be unnecessary. The real characteristic of malignant hepatic PEComa is clinical evidence of aggressive behavior such as death owing to disease or metastasis. It appears that longer clinical follow-up is necessary in all patients with hepatic PEComas because the nature of the disease is not entirely known at present. In the future, the origin, differentiation, function, and distribution of the disease need to be explored, and the criteria that accurately predict the behavior of PEComa should be established. The establishment of hepatic PEComa online clinical register seems to be necessary.

## Acknowledgment

We thank the Pathology Department of the Second Xiangya Hospital which provided the histological and immunohistochemical figures for the authors, and this department gives permission to be named.
